# Skin Diseases and Syphilis

**Published:** 1893-09

**Authors:** 


### SKIN DISEASES AND SYPHILIS.

---

EDITED BY DR. M. B. HUTCHINS.

---

### CAVERNOUS NÆVI.

At the meeting of the Berlin Dermatological Society, March 14th, Peter presented a case of a child with a monstrous cavernous nævus, involving almost the entire face. For the cure of these in children, on circumscribed places, a crust was made which continued until (as in another case) a smooth white scar had taken its place, after two months. The crusts are formed partly by concentrated carbolic acid, partly by Paquelin cautery.—*Monatshafte für Prak. Derm., XVII., No. 1.*

---

### PIGMENT SPOTS UPON THE SKIN DURING PREGNANCY.

*Jour. des Mal. Cut. et Syph.*, 1892, p. 728, has following salve as recommended for the removal of these pigmentations:

℞. Zinc oxid. pur., gr. v.
Hydrarg. ox. flav., gr. xx.
Ol. ricini,
Ol. theobromat., aa. ℥iiss.
Spirit. ros., gtt. x.
M. Sig.: Rub in the affected points b. i. d.

*M. für Prak. Derm., XVII., No. 1.*

---

### THE QUESTION OF THE CURABILITY OF EPITHELIOMA.

O. Lassar discussed this question in the Berlin Medical Society, demonstrating with patients, drawings and microscopical preparations. The speaker had already, 1889, brought a case of multiple carcinoma of the skin near to cure by therapeutic means. Lately he had followed the method with another patient, who had a walnut-sized tumor of the skin of the cheek—microscopically shown to be flat celled "skin cancer." The tumor had cicatrized without local treatment. A second case is getting well. The treatment was with arsenic internally or subcutaneously (hypodermatically). "This arsenic treatment is worth further trial."—(*Berl. Klin. Wochenschr*) *M. für P. D., XVII., No. 1.*

[This method of treatment has been highly spoken of for the cure of skin sarcoma. The question is whether (carcinoma and sarcoma being such different processes) the drug simply acts upon elements common to both, or whether it really has a specific effect upon the two kinds of different cell growths in the two classes of disease. —ED.]

---

### THE OCCURRENCE OF TERTIARY LESIONS OF SYPHILIS AS THE RESULT OF DIRECT LOCAL INFECTION.

In the July and August numbers of *The Journal of Cutaneous and Genito-Urinary Diseases*, Dr. Hermann G. Klotz discusses the possibility of tertiary lesions of syphilis being contracted by direct infection from these lesions on another, without the victim having passed through the preceding stages. The article is fuller of theory than of logical proof. His argument rests upon those experiences we all have of the appearance of tertiary lesions upon persons who have no history of the preceding stages of syphilis; he mentions two (doubtful) cases of his own where the contagion seemed direct. Much of his reasoning is an attempt at the demonstration of an analogy between local tubercular processes and tertiary syphilides, but in the case of the latter, the reasoning is based upon suppositious grounds. When actual experience and scientific research prove the correctness of the doctor's views (if they do) he can, at least, have the credit for having anticipated them.

---

### SARCOMA OF THE TONGUE.

Scheier (Berlin Laryng. Soc.) spoke of a sarcoma of the base of the tongue the diagnosis of which could only be made through microscopic examination of small pieces repeatedly taken from the growth. "Radical removal" was quickly followed by recurrence.

The disease is very rare, the speaker having found only fourteen cases in literature. The position is chiefly in the base of the tongue. Rapidity of growth variable. In diagnosis, gummata and carcinoma are to be considered. Prognosis unfavorable. In one case the tumor was congenital, and was successfully removed with the galvano-cautery.—*Monatsh. für Prak. Derm., XVII., No. 1.*

[A case of sarcoma of the tongue was in Atlanta some months ago. A young man was advised by those he consulted that an operation offered no hope.—ED.]

---

### THE SO-CALLED "PEELING METHOD" FOR FRECKLES, ACNE ROSACEA, ETC.

W. Van Hoorn (*Progres Medical, 1893, No. 4*) had employed, with best results, Unna's "scaling method," by means of 50 per cent. resorcin paste, followed by "zinc lime," for freckles. He was thus able to remove in one sheet the epidermis receiving the application. Upon the under surface of the sheet the freckles were apparent as brown spots, the comedones of the nasal region as small points.—*Monatsh. für P. D., XVII, No. 1.*

[The only trouble about removing freckles by removing the epidermis is that they soon recur in the new formed epidermis.—ED.]

---

### A NEW METHOD OF TREATING IDIOPATHIC BALDNESS.

Morel-Lavallee employs scarification as a means of bringing back the hair on bald spots. (Of course the hair papillae must still remain.) He cleans and disinfects the spot, and then scarifies it superficially. Then "a salve is applied for twelve to twenty-four hours. In five to eight days the operation is repeated. Other procedures unnecessary."

Eight days after the second, or at furthest, eight days after the third, operation the hairs are beginning to return, seeming to grow from edge to center in order.—*Exchange.*

[As Morrow says, the treatment of baldness is summed up in the word *stimulation.*—ED.]

### PREMATURE BALDNESS.—ABSURDITIES.

*Monatsheft für Prak. Derm.* refers to an article by Seeger upon this subject. He revives the fanciful theory that premature baldness is more common in men because of the compression of the hat shutting off circulation, and, perhaps, innervation also. A moment's thought on the subject would seem to disprove the correctness of this reasoning. The blood and nerve supply of all men's scalps are the same. They all wear hats. They do not all get bald. He thinks hats make the scalp too hot, increase sweating there, the sweat is not allowed to evaporate, causes less activity and hair growth, the reaction comes—the hairs cease to be formed. If pressure disturbs or shuts off the circulation and innervation, how can increased (even temporary) activity occur?

"Mental work" hastens the fall of hair, keeping the man indoors out of the fresh air. A man engaged in indoor mental work doesn't usually keep his hat on, so the only chance is to stay outdoors and go bareheaded.

As a part of the treatment the hair must be worn so long that every one sticks out beneath the hat. (A man would as leave be baldheaded as "mop-headed.") Another thing to do is to vigorously agitate the hair by means of the occipito-frontalis muscle.

[There are a few good points in the article, but there are so many absurd ones that we are unwilling to accept any. Certain self-supposed writers and investigators are constantly filling medical publications with a lot of unscientific theories which have absolutely no foundation in anatomy, philosophy or reason.—ED.]

---

NEVER give stimulants in a case of profuse hemorrhage. The faint feeling, or irresistible inclination to lie down, is nature's own method of circumventing the danger, by quieting the circulation and lessening the expulsive force of the heart, thus favoring the formation of clot at the site of injury.—*Clinique.*

